# Supplementary material for: Adherence to the Healthy Eating Index-2015 across Generations Is Associated with Birth Outcomes and Weight Status at Age 5 in the Lifeways Cross-Generation Cohort Study
Source: Nutrients. 2019 Apr 25;11(4):928. doi: 10.3390/nu11040928 (PMC6520851; doi:10.3390/nu11040928)
Supplement: Supplementary file 1 [file nutrients-11-00928-s001.zip › Figure S1. Participant flow chart_Nutrients.docx]

**Figure S1.** Participant flow chart in the current analysis of the Lifeways study

Recruited mothers initially (n=1132)

Incomplete baseline questionnaire (n=8)

Recruited mothers (n=1124)

Available families at birth (n=1082)

Live born infants with 12 twins (n=1094)

Singleton mothers with FFQ data (n=1082)

Fathers with FFQ data (n=333)

Grandparents with FFQ data (n=707)

Miscarriages/stillbirth (n=23)

Maternal death (n=1)

Missing hospital record or born at other hospital (n=14)

Withdrawal before birth (n=4)
