# Supplementary material for: Adherence to the Healthy Eating Index-2015 across Generations Is Associated with Birth Outcomes and Weight Status at Age 5 in the Lifeways Cross-Generation Cohort Study
Source: Nutrients. 2019 Apr 25;11(4):928. doi: 10.3390/nu11040928 (PMC6520851; doi:10.3390/nu11040928)
Supplement: Supplementary file 1 [file nutrients-11-00928-s001.zip › Table S2.docx]

| **Table S2.** Associations between grandparental HEI scores and continuous offspring outcomes at birth and at five years^1^ | | | | | | | | | | |
| --- | --- | --- | --- | --- | --- | --- | --- | --- | --- | --- |
|  | **Birth weight, g** | | **Birth length, cm** | | **Head circumference, cm** | | **Birth BMI, kg/m^2^** | | **BMI at 5 years, kg/m^2^** | |
| ***HEI scores*** | **Unadjusted** | **Multivariable** | **Unadjusted** | **Multivariable** | **Unadjusted** | **Multivariable** | **Unadjusted** | **Multivariable** | **Unadjusted** | **Multivariable** |
| ***MGM*** |  |  |  |  |  |  |  |  |  |  |
| T2 | 0.10  (-0.12, 0.17) | 0.12  (-0.01, 0.44) | -0.02  (-0.10, 0.42) | -0.12  (-0.21, 0.71) | 0.03  (-0.11, 0.25) | 0.13  (-0.09, 0.43) | 0.08  (-0.04, 0.35) | 0.16  (-0.06, 0.36) | -0.18  (-0.39, 0.24) | -0.17  (-0.25, 0.25) |
| T3 | 0.25 (-0.13, 0.41) | **0.21  (0.02, 0.35)*** | -0.09  (-0.15, 0.23) | -0.27  (-0.37, 0.68) | 0.02  (-0.13, 0.30) | 0.09  (-0.08, 0.44) | 0.12  (-0.13, 0.30) | **0.11**  **(0.01, 0.24)*** | -0.06  (-0.23, 0.17) | -0.12  (-0.31, 0.20) |
| Cont. | 0.05 (-0.01, 0.10) | **0.13 (0.05, 0.30)*** | **-0.04**  **(-0.07, -0.01)*** | -0.10  (-0.02, 0.25) | 0.10  (-0.03, 0.32) | 0.12  (-0.01, 0.32) | 0.07  (-0.06, 0.20) | **0.08**  **(0.04, 0.20)*** | -0.02  (-0.10, 0.05) | -0.07  (-0.18, 0.05) |
| *P*_trend_ | 0.26 | **0.01** | **0.03** | 0.11 | 0.09 | 0.11 | 0.21 | **0.02** | 0.62 | 0.21 |
| ***MGF*** |  |  |  |  |  |  |  |  |  |  |
| T2 | 0.12  (-0.07, 0.30) | 0.16  (-0.17, 0.20) | -0.54  (-0.86, 0.86) | -0.39  (-0.62, 0.24) | 0.23  (-0.58, 0.85) | 0.41  (-0.27, 0.55) | 0.15  (-0.28, 0.47) | 0.13  (-0.45, 0.31) | 0.24  (-0.16, 0.80) | 0.21  (-0.20, 0.95) |
| T3 | 0.05  (-0.16, 0.21) | 0.10  (-0.11, 0.28) | -0.27  (-0.51, 0.78) | -0.29  (-0.43, 0.21) | 0.22  (-0.30, 0.73) | 0.30  (-0.26, 0.80) | 0.19  (-0.19, 0.45) | 0.23  (-0.41, 0.72) | -0.15  (-0.72, 0.25) | 0.07  (-0.55, 0.86) |
| Cont. | 0.03  (-0.04, 0.14) | 0.04 (-0.06, 0.18) | -0.43  (-0.58, 0.32) | -0.40  (-0.84, 0.77) | 0.28  (-0.25, 0.91) | 0.30  (-0.10, 0.89) | 0.21  (-0.48, 1.08) | 0.16  (-0.39, 0.89) | -0.20  (-0.59, 0.73) | -0.73  (-0.80, 0.99) |
| *P*_trend_ | 0.15 | 0.24 | 0.14 | 0.25 | 0.49 | 0.38 | 0.63 | 0.70 | 0.31 | 0.66 |
| ***PGM*** |  |  |  |  |  |  |  |  |  |  |
| T2 | -0.02  (-0.24, 1.12) | 0.07  (-0.41, 0.78) | -0.12  (-0.31, 0.41) | 0.03  (-0.11, 0.42) | -0.15  (-0.21, 0.65) | -0.14  (-0.30, 0.81) | 0.23  (-0.12, 0.84) | -0.06  (-0.25, 0.88) | -0.21  (-1.05, 0.23) | 0.10  (-0.22, 0.46) |
| T3 | 0.04  (-0.21, 1.04) | -0.08  (-0.31, 1.10) | 0.10  (-0.02, 0.43) | 0.12  (-0.14, 0.76) | -0.13  (-0.44, 0.47) | -0.11  (-0.21, 0.85) | 0.41  (-0.23, 0.72) | 0.05  (-0.35, 0.64) | -0.76  (-1.10, 0.38) | -0.24  (-0.90, 0.91) |
| Cont. | 0.11  (-0.12, 0.87) | 0.13  (-0.15, 0.89) | 0.27  (-0.08, 0.51) | 0.59  (-0.02, 0.87) | -0.09  (-0.27, 0.78) | -0.15  (-0.47, 0.95) | 0.54  (-0.19, 0.87) | 0.09  (-0.41, 0.68) | -0.87  (-1.23, 0.25) | -0.41  (-1.25, 0.54) |
| *P*_trend_ | 0.12 | 0.27 | 0.24 | 0.27 | 0.72 | 0.61 | 0.74 | 0.49 | 0.26 | 0.25 |
| ***PGF*** |  |  |  |  |  |  |  |  |  |  |
| T2 | -0.09  (-0.12, 0.58) | -0.14  (-0.21, 0.69) | 0.10  (-0.05, 0.48) | 0.25  (-0.12, 0.94) | 0.04  (-0.24, 0.69) | -0.12  (-0.78, 0.41) | 0.07  (-0.73, 0.48) | -0.12  (-0.37, 0.80) | 0.26  (-1.11, 0.88) | 0.10  (-0.29, 0.99) |
| T3 | 0.08  (-0.04, 0.41) | 0.15  (-0.11, 0.40) | 0.21  (-0.03, 0.77) | -0.15  (-0.32, 1.14) | -0.88  (-1.28, 0.27) | -0.63  (-1.26, 0.17) | -0.05  (-0.40, 0.74) | -0.16  (-0.55, 0.71) | 0.16  (-0.79, 1.10) | -0.15  (-1.01, 0.95) |
| Cont. | -0.06  (-0.46, 0.12) | -0.02  (-0.28, 0.31) | -0.16  (-1.02, 0.52) | -0.52  (-0.73, 0.21) | -0.98  (-1.18, 0.18) | -1.07  (-1.30, 0.11) | -0.03  (-1.01, 0.86) | -0.25  (-1.44, 0.84) | 0.32  (-1.14, 1.20) | -0.22  (-1.82, 1.11) |
| *P*_trend_ | 0.62 | 0.47 | 0.48 | 0.15 | 0.19 | 0.30 | 0.25 | 0.24 | 0.68 | 0.44 |
| ^1^Values are β (95% CI) with reference to the lowest tertile (T1) for tertile analysis. Multivariable models were adjusted for socio-economic status, education status, marital status, cigarettes smoking and alcohol consumption, household income, marital status, age at recruitment, body mass index, and child age at follow-up (included in models for 5yr outcomes only) and sex (for overweight and obese status child sex was intrinsically adjusted). Missing covariate information was handled by pooling effect estimates from 20 multiply-imputed datasets. *p<0.05 | | | | | | | | | | |
